# In vitro surface analysis of the brushing resistance of orthodontic sealants using two different profilometric evaluation methods

**DOI:** 10.1038/s41598-022-19702-7

**Published:** 2022-09-27

**Authors:** J. Lorenz, I. Schidtmann, M. Morawietz, A. Kiesow, H. Wehrbein, S. Sarembe, C. Erbe

**Affiliations:** 1 Department of Orthodontics, University Medical Center of the Johannes Gutenberg-University, Augustusplatz 2, 55131 Mainz, Germany; 2 Fraunhofer Institute for Microstructure of Materials and Systems IMWS, Characterization of Medical and Cosmetic Products, Halle, Germany; 3 Institute for Medical Biostatistics, Epidemiology and Informatics (IMBEI), University Medical Centre of the Johannes Gutenberg-University, Mainz, Germany

**Keywords:** Caries sealants, Fixed appliances, Plaque

## Abstract

The enamel can be protected by applying orthodontic sealants at the bracket base to avoid the development of white spot lesions caused by inadequate oral hygiene. The aim of this study was to investigate the mechanical resistance of five commonly used orthodontic sealants against brushing in comparison to a positive group. Hydroxyapatite discs were bonded with a metal bracket and a piece of arch-wire was ligated in order to simulate a daily clinical situation (n = 48). Samples were divided into 6 groups of respectively 8 specimens. Sealants were applied around the bracket base according to manufacturer’s instructions. Following sealants were used: Group 1: Pro Seal (Reliance Orthodontic Products, Itasca, Illinois, USA); 2: Light Bond (Reliance Orthodontic Products, Itasca, Illinois, USA); 3: ClinproXT Varnish (3M ESPE, Seefeld, Germany); 4: ProtectoCaF2 Nano (BonaDent GmbH, Frankfurt am Main, Germany); 5: Fluor Protector and 6: Tetric EvoFlow (both Ivoclar Vivadent AG, Schaan Liechtenstein). Tooth brushing were simulated for 6 weeks and 6 months with an electric toothbrush. The sealant thickness was measured by mechanical (MP) and optical profilometry (OP) at baseline, after 6 weeks and after 6 months of brushing. Statistical analysis was performed according to two mixed linear models and post hoc Tukey–Kramer comparisons. The significance level was set at 5% (α ≤ 0.05). Pro Seal (MP: 9%; OP: 22%) and Light Bond (MP: 19%; OP: 16%) showed the lowest changes in sealant thickness after 6 months of simulated brushing. ClinproXT Varnish and Tetric EvoFlow recorded no statistically significant results (*p* > 0.05). The fluoride varnishes ProtectoCaF2 Nano and Fluor Protector could not be conclusively evaluated since the thickness of the sealants could not be determined at baseline. The results of both evaluation methods MP and OP are in good agreement. Pro Seal and Light Bond were resistant against tooth brushing and were able to adequately keep the bracket environment sealed even after 6 months. The two different measuring methods, MP and OP, provide a precise impression of the changes in the surface.

## Introduction

Fixed orthodontic appliances are used worldwide to correct malocclusions. During this treatment, teeth are exposed to an increased risk of caries and demineralization, requiring patients to maintain above-average oral hygiene. A frequently observed, undesirable side effect is the appearance of white spot lesions (WSL)^1^. These are white, chalky spots on the enamel caused by initial demineralization. When WSL are active, they represent initial carious lesions. Risk factors to developing WSL include inadequate oral hygiene and prepubescent age at the beginning of treatment^2^.

Prophylactically, the enamel surrounding the bracket area, which is particularly difficult to clean, can be protected during the orthodontic treatment by applying sealants at the bracket base directly after bracket bonding. The current common sealant methods are fluoride varnishes applied directly onto the tooth and fluoride-composite filled sealants applied with the acid etch technique and photo polymerization. These products are expected to provide caries prevention and protection against external stress between 6 and 12 months. Various studies demonstrate the protective effect of sealants^3–5^ and describe a significant reduction in the average lesion depth of the adjacent enamel^3,6^.

The aim of this study was to determine how the sealants thickness change after daily mechanical load of tooth brushing, and to determine whether one can guarantee an effective seal at 6 weeks and at 6 months after application. Five commonly used sealants were investigated in this study in comparison to a positive control. Two evaluation methods (mechanical and optical profilometry) were tested and compared to each other.

## Material and methods

### Sample preparation

Five most commonly used sealants reported in a survey of 985 dentists from orthodontic practices in Germany were investigated in this study^7^.

Forty-eight HA discs were divided into six experimental groups:

- Group 1: Pro Seal (Reliance Orthodontic Products, Itasca Illinois, USA).
- Group 2: Light Bond (Reliance Orthodontic Products, Itasca Illinois, USA).
- Group 3: ClinproXT Varnish (3M Unitek, Landsberg, Germany).
- Group 4: ProtectoCAF2 Nano (BonaDent GmbH, Frankfurt am Main, Germany).
- Group 5: Fluor Protector (Ivoclar Vivadent AG, Schaan, Liechtenstein).
- Group 6: Positive Control: Tetric EvoFlow (Ivoclar Vivadent AG, Schaan, Liechtenstein).

HA discs (Clarkson Chromatography Products, Inc., South Williamsport, PA USA) were used as flat enamel substitutes. All materials were used strictly according to the manufacturer's instructions. All HA discs were cleaned with fluoride-free cleaning and polishing paste (Zircate Prophy Paste, DENTSPLY DeTrey GmbH, Constance, Germany), sprayed with water and dried. After conditioning the surface with 35% phosphoric acid (Etching Gel, 3M Unitek), a nickel-free Mini-Sprint bracket made of stainless steel (Forestadent^®^, Pforzheim Germany) was bonded to each HA disc. The adhesive systems, Transbond XT Light Cure Adhesive Primer and Transbond XT Light Cure Adhesive Primer (both 3M Unitek) were used. After bracket bonding, the HA discs were cleaned again with the fluoride-free cleaning and polishing paste (Zircate Prophy Paste) to remove adhesive residue. The HA discs were numbered consecutively and masked with an adhesive strip (tesa Global Headquarters—tesa SE, Norderstedt, Germany) so that only the area around the bracket could be sealed and a smooth transition was created between the varnish layer and the HA. The reference material and sealants were then applied.

Before each brushing interval, an orthodontic single arch (1 cm) with a cross-section of 0.016″ × 0.022″ inch (Forestalloyblue, Forestadent^®^, Pforzheim, Germany) was ligated into the bracket with an elastic (Dentalastics^®^Personal, DENTAURUM GmbH & Co. KG, Ispringen, Germany).

### Brushing treatment

**Figure 1:**
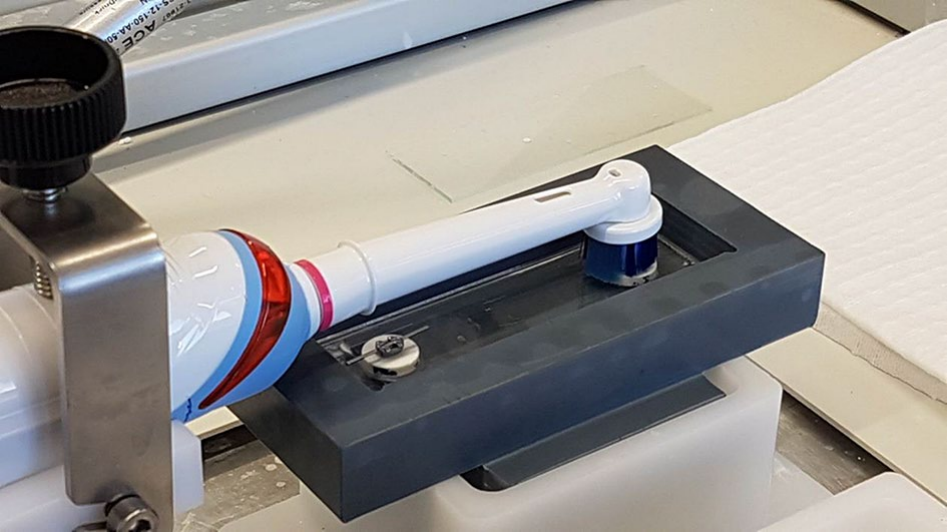
Test set-up of the brushing treatment using a brushing simulator.

The experimental test set-up is depicted in Fig. 1. To mimic the mechanical stress of tooth brushing, the specimens were brushed using the ZM-3.8 toothbrush simulator (SD-Mechatronik, Feldkirchen-Westerham, Germany). The Oral-B PRO 1000 electric toothbrush (Procter & Gamble Service GmbH, Schwalbach am Taunus, Germany) with the Precision Clean brush head was used. The brush head was renewed, and the battery fully recharged after every brushing procedure and sample. 0.3–0.4 g of elmex toothpaste (CP GABA GmbH, Hamburg, Germany) was added onto the moistened brush head before brushing.

A zig-zag motion was selected, with a linear brushing path of 10 mm and a lateral brushing path of 8 mm (brushing speed 30 mm/s). The contact force of the brush head was set to 2N and controlled by the included contact pressure control of the toothbrush.

Brushing times were adopted from Deckers et al.^7^. The first brushing interval was 126 s and simulated a brushing duration of 6 weeks. For the second brushing interval, the samples were additionally brushed for 378 s simulating a total brushing duration of 6 months (total brushing time 504 s).

Calculation of the brushing times: A two-minute brushing time was assumed for each tooth cleaning and a fully edentulous set of 28 teeth. The brushing time per quadrant is 30 s and 4.3 s per tooth. With three tooth surfaces that can be reached with the toothbrush (buccal, occlusal, and oral), the brushing time per surface corresponds to 1.4 s. For the calculation of the total time, an average brushing time of 1.5 s per tooth surface and brushing procedure is assumed. With twice-daily cleaning, each tooth surface is brushed for 3 s.

Assuming 6 weeks (42 days), this results in a brushing time of 126 s.

Assuming 6 months (180 days), this results in a brushing time of 504 s.

### Surface analysis

**Figure 2:**
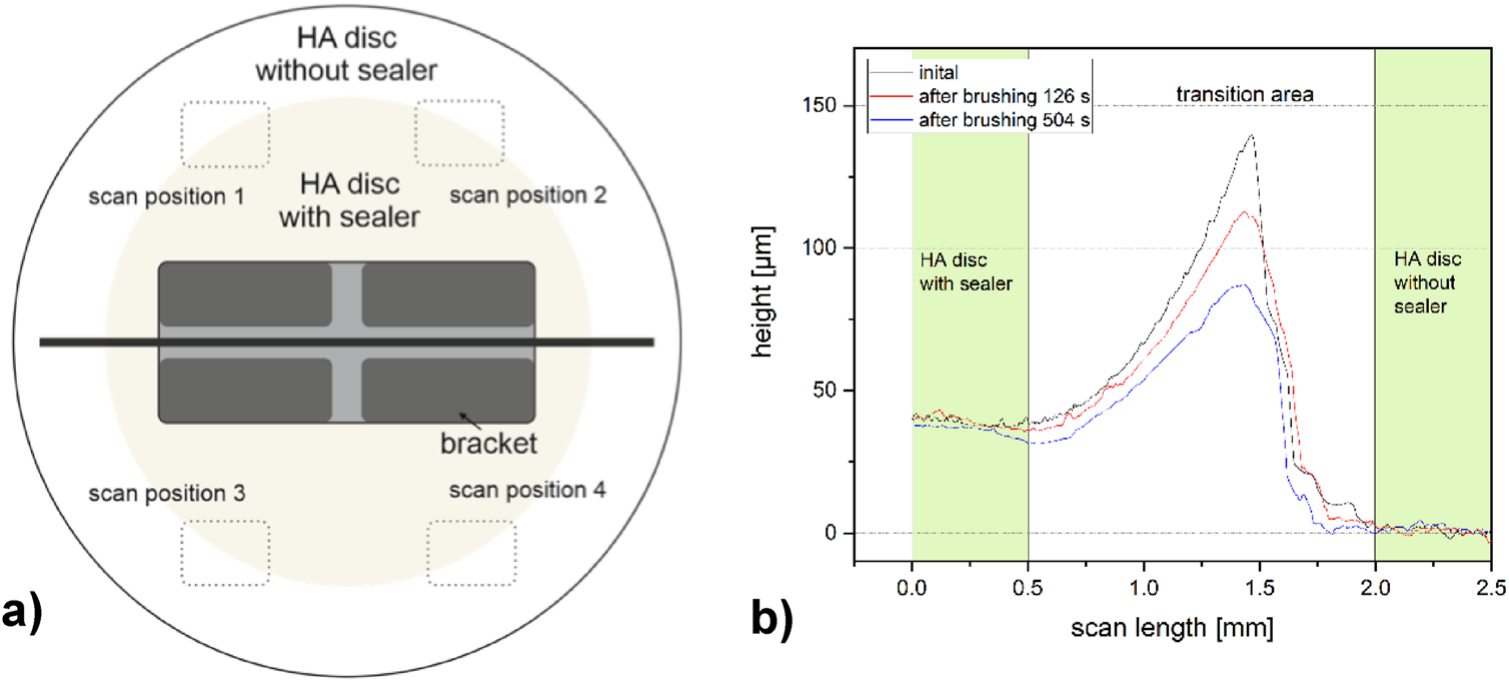
(**a**) Schematic drawing of a hydroxyapatite disc including the measuring positions. (**b**) Example of a profile image determined by mechanical profilometry.

Before and after each brushing step, the thickness of the sealants was examined using two different measurement techniques, MP and OP. A schematic drawing of the samples including the four measuring points (see dashed lines) is depicted in Fig. 2a. The difference in height between the HA discs with sealant and the reference area (HA disc without sealant) was determined in the areas highlighted in green (Fig. 2b). The green measurement windows were set at representative points per sample.

The Dektak XT stylus profilometer (Bruker Corporation, Billerica, Massachusetts, USA) was used for MP. A diamond probe (tip radius 2 µm, tip angle 60°) moves tactilely along the sample surface. The automated and integrated software (Vision64) converted the analog data determined by the vertical deflection of the probe into digital data. This creates height and thickness profiles of the sealants.

OP involves a laser scanning microscope (Leica Microsystems GmbH, Wetzlar, Germany) used to create 3D scans of the surface topographies (area profile of the change in height). This method makes it possible to obtain qualitative and quantitative information on the changes in layer thickness. Therefore, each sample was scanned using a 633 nm laser light and 10× objective lens.

Data was collected using Microsoft Excel 2010 and bar charts were created using Microsoft Excel 2016 (both Redmond, Washington, USA). The baseline measurements were recorded for each sample, at the 6-week, and 6-month time points. The mean and standard deviation of the material loss, i.e., change of layer thickness [%], was calculated using the following equation:

$${\text{change}}\;{\text{of}}\;{\text{thickness}}\left[\% \right] = \frac{{sealer\;thickness_{after\;brushing} - sealer\;thickness_{initial} { }}}{{sealer\;thickness_{initial} }}$$

Statistical analysis was performed using a mixed linear model. One model was created for the layer thickness at the three measurement times (at the baseline, after brushing time 1, after brushing time 2). The second model represented the change in coating thickness after 6 weeks and 6 months compared with the initial coating generated. The respective sealant, the measuring method and the measuring time were fixed effect parameters. The significance level of the statistical evaluation of the results from this study were set at 5% (α ≤ 0.05). Tukey–Kramer post-hoc tests were performed for multiple comparisons of the adjusted means.

### Study design

**Figure 3:**
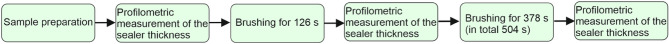
Flow diagram to illustrate the experimental approach.

For better illustration, the following flow diagram represents the experimental procedure (see Fig. 3).

### Ethics approval and consent to participate

Not applicable. No human subjects were involved in this study.

## Results

### Mechanical profilometry

**Figure 4:**
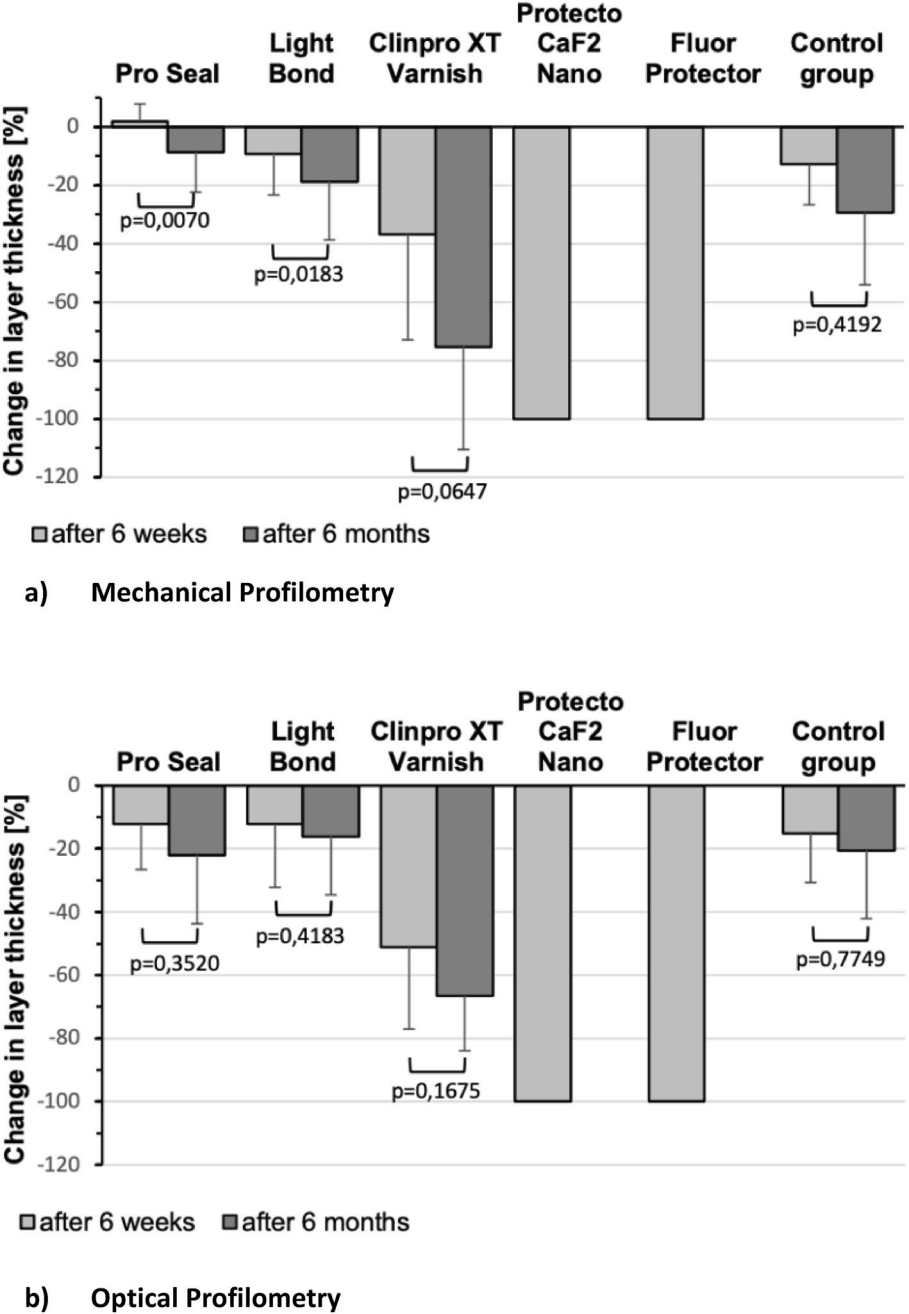
Diagram showing profilometry comparison of all sealants; light grey bars represent the measuring points after 6 weeks; dark grey bars represent 6 months: (**a**) mechanical profilometry, (**b**) optical profilometry. There are no dark grey bars for Protecto CAF2 and Fluor Protector because both were completely removed after 6 weeks, therefore no longer measurable.

The results of the mechanical profilometry are depicted in Fig. 4a. The mean percent reduction of sealant layer thickness (material loss) of Pro Seal is 2.0% ± 5.9% at 6 weeks and 8.7% ± 13.7% at 6 months. These values are statistically significant (*p* = 0.0070) comparing mean values after 6 weeks and 6 months. The mean percent reduction of sealant layer thickness of Light Bond is 9.2% ± 14.1% at 6 weeks and 18.7% ± 20.0% at 6 months. The material loss is statistically significant (*p* = 0.0183) comparing mean values after 6 weeks and 6 months. A mean percent material loss of 36.8% ± 36.1% after the first brushing interval and 75.3% ± 35.3% after the second brushing interval was recorded for ClinproXT Varnish. The mean change in thickness is not statistically significant (*p* = 0.0647) comparing mean values after 6 weeks and 6 months. The mean percent material loss of the control group Tetric EvoFlow is 12.8% ± 13.9% after 6 weeks and 29.3% ± 24.7% after 6 months and is not statistically significant different (*p* = 0.4192).

### Optical profilometry

Figure 4b depicts the change of the layer thickness measured by OP. The mean percent reduction of the sealant layer thickness (material loss) of Pro Seal is 12.1% ± 14.4% at 6 weeks and 22.0% ± 21.7% at 6 months. The amount of material loss is not statistically significant (*p* = 0.3520) comparing mean values after 6 weeks and 6 months. The mean percent reduction of sealant layer thickness of Light Bond is 12.2% ± 20.0% at 6 weeks and 16.1% ± 18.5% at 6 months. The material loss is not statistically significant (*p* = 0.4183) comparing mean values after 6 weeks and 6 months. ClinproXT Varnish recorded a mean percent reduction of sealant layer thickness of 51.1% ± 26.0% after the first brushing interval and 66.6% ± 17.3% after the second brushing interval. The change of material loss is not statistically significant (*p* = 0.1675) comparing mean values after 6 weeks and 6 months. The mean percent reduction of sealant layer thickness of the control group Tetric EvoFlow is 15.1% ± 15.6% after 6 weeks and 20.5% ± 21.5% after 6 months. The change in material loss is not statistically significant (*p* = 0.7749) comparing mean values after 6 weeks and 6 months.

Due to the low initial layers thickness, the sealants ProtectoCaF2 Nano and Fluor Protector could not be measured by both applied methods.

**Figure 5:**
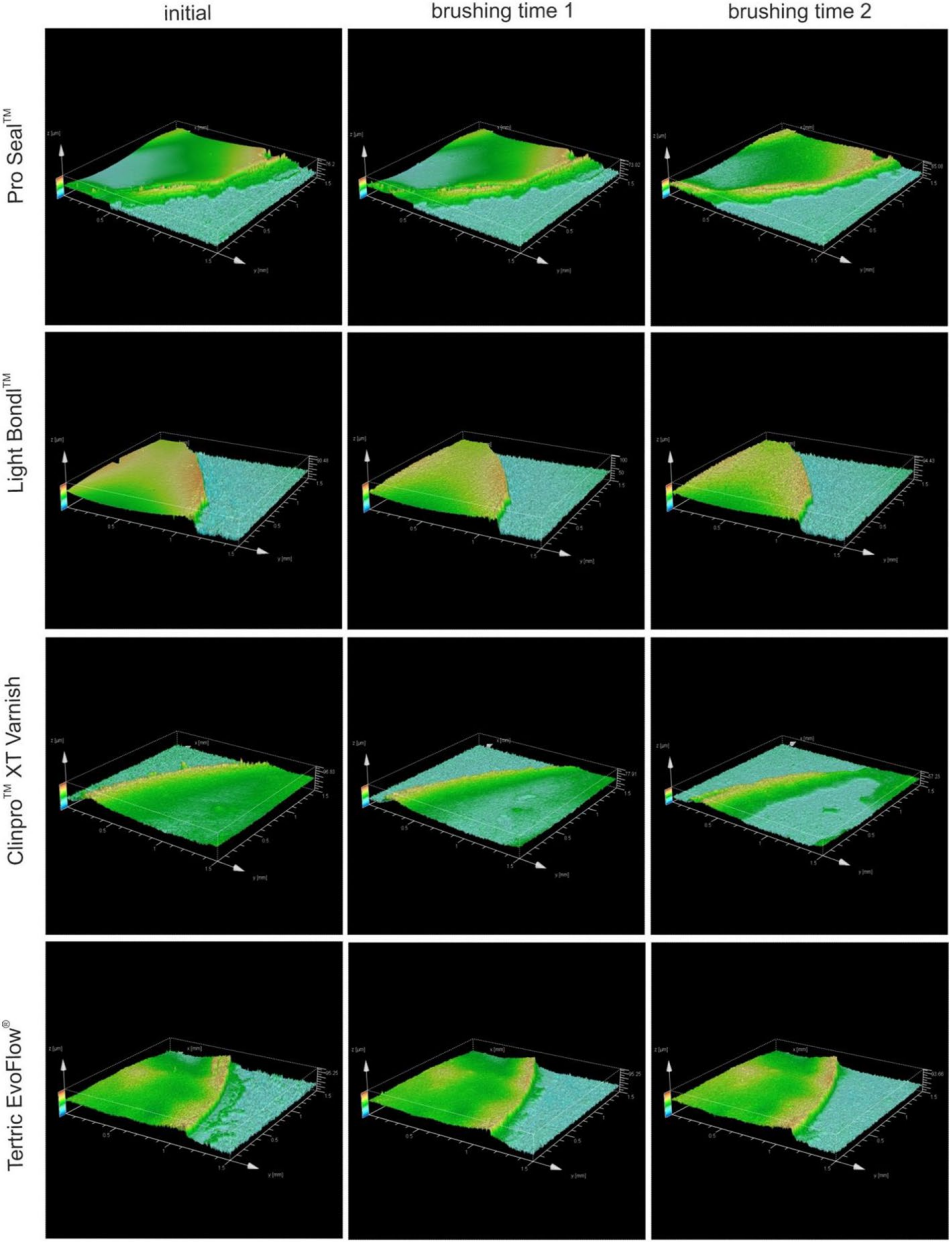
3D images of the sealants surface topography of Pro Seal, Light Bond, ClinPro and Tetric EvoFlow determined by optical profilometry.

**Figure 6:**
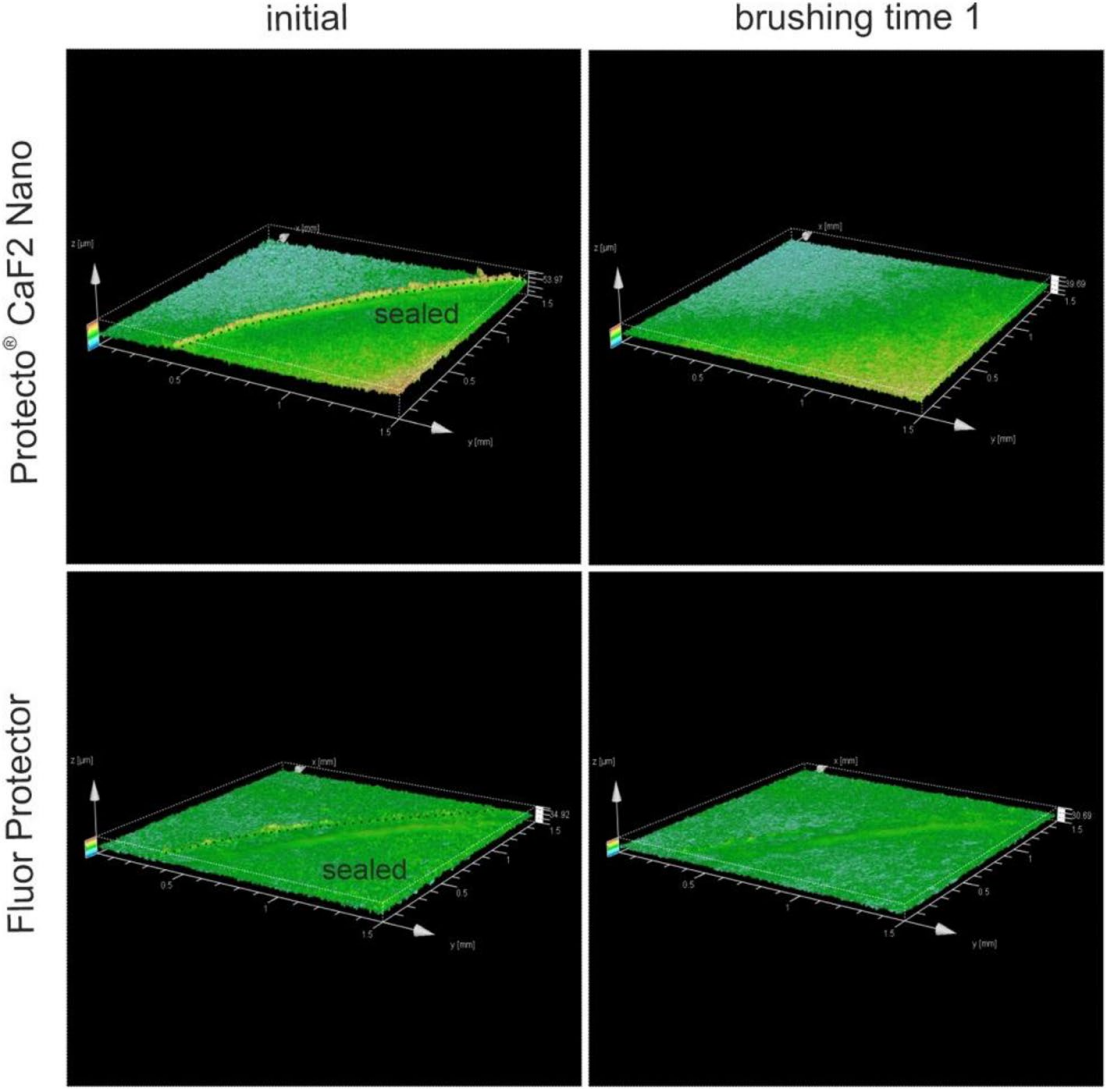
3D images of the sealants surface topography of ProtectoCaF2 Nano and Fluor Protector determined by optical profilometry.

Furthermore, 3D images of the surface topography were recorded. A selection of images of Pro Seal, Light Bond, ClinPro and Tetric EvoFlow are depicted in Fig. 5 at baseline and after both brushing times. All images show a smoother transition after both rendering processes. Images of the ProtectoCaF2 Nano and Fluor Protector show that the sealants are very thin at the baseline and are removed after the first brushing step (Fig. 6). Therefore, the second brushing step was not performed.

### Comparison of the results determined by mechanical and optical profilometry

Considering the material loss, no statistically significant measurement method effect can be detected overall (*p* = 0.9386). The mean level is similar across all measurement methods. The time effect *p* = 0.0002 is statistically significant. This means that the wear is different at the two measurement times. However, the time effect is independent of the measurement method and the sealant. All other mentioned results are not statistically significant (method*time: *p* = 0.1908; coating*time: *p* = 0.3086; method*coating*time: *p* = 0.9163).

## Discussion

HA discs have already been used to replace enamel surfaces in past studies^8–12^. They are produced in a standardized manner, all have an identical surface texture, and are unaffected by external influences as they exist in the oral cavity prior to extraction. In the study by Imthiaz et al.^13^, HA discs and human enamel were compared, and it was concluded that HA discs can be used as an alternative to enamel in comparative laboratory studies. Each disc was bonded with a conventional metal bracket and a piece of arch-wire was ligated in order to simulate a daily clinical situation.

MP and OP are measurement methods widely used in the literature, and are frequently reported techniques in the study of tooth surfaces^14–21^. The measuring positions of the profilometric investigations on the HA disc (transition from sealed and unsealed area) were individually selected by the examiner for each specimen and were not identical for MP and OP. Thus, possible changes of the sealant caused by the contact of the stylus during the MP measurement cannot influence the optical measurement. Overall, there are significant differences between the sealant thickness values when comparing both measurement methods. However, when the change of the sealants thickness is considered, as is sufficient for the purpose of this study, this method effect does not apply. The significantly different level of coating thickness is largely cancelled out by the difference. Therefore, one measurement method is sufficient for studies of this type.

There are a wide range of sealants available on the dental market. The literature contains numerous in vitro as well as in vivo studies due to sealing the area around the bracket base. In several studies Tetric EvoFlow has shown a good protective effect against abrasive and erosive influences, as well as longevity^7,22–24^ and has therefore been selected as the positive control group/ material in this study.

The manufacturer of Pro Seal promises "protection over the entire treatment period". Many studies describe comparable results and list Pro Seal as the best of all the materials tested. In 2011, Shinaishin et al.^25^ conducted an in vivo study on the effectiveness of light-activated sealants in regards to protection against enamel demineralization. The Pro Seal group achieved the lowest degree of roughness and the lowest total surface area. The characteristics of the sealant surface structures were comparable to those of ordinary human enamel. Based on these results, the use of Pro Seal was concluded to be an efficient prophylactic measure to reduce the development of enamel demineralization. The results of the in vitro study by Buren et al.^26^ also confirm that Pro Seal can reduce the average lesion depth by up to 97% compared to the control group. Hu and Featherstone^27^ demonstrate that after mechanical and chemical loading, Pro Seal records significantly less material loss than the other sealants.

Deckers et al.^7^ investigated similar sealants to those tested in this study. They were applied to extracted bovine teeth in the bracket environment and subjected to mechanical, chemical and thermal stress. The material defects of Pro Seal were the lowest of all the materials tested, being comparable to the control group Tetric EvoFlow. In this study, the lowest material loss of the sealants tested was recorded with Pro Seal. It was 2.0% (MP) and 12.1% (OP) after 6 weeks and 8.7% (MP) and 22.0% (OP) after 6 months. These values testify to a high mechanical load-bearing capacity of the material used.

In 2009, Tanna et al.^28^ investigated in vitro the effect of sealants and self-conditioning primers on enamel demineralization. Enamel lesions were observed in 50% of the Light Bond samples, with an incidence of 100% in the primer and control groups. Heinig and Hartmann^29^ confirmed the protective properties of Light Bond against enamel demineralization in their clinical study on the effectiveness of a sealant. In contrast to the unsealed teeth, significantly fewer areas of shallower depth were affected by demineralization. In 2013, Korbmacher-Steiner et al.^30^ investigated the abrasion resistance of four different sealants to toothbrush and toothpaste in vitro. In the case of LightBond, defects in the sealant layer with a diameter of up to 300 µm were observed after 2 years. Bechtold et al.^31^ investigated Light Bond in a clinical study in 2013. No caries-protective effect was observed after 6 months. Deckers et al.^7^ recorded a significant material loss of 38.4% after 6 weeks and 39.7% after 6 months in his study. These values are significantly higher than the results obtained in the present study for the change in layer thickness (i.e. material loss). In the current study^7^, the sealant Light Bond achieved a higher overall material loss in comparison to Pro Seal. This was 9.2% (MP) and 12.2% (OP) after 6 weeks and 18.7% (MP) and 16.1% (OP) after 6 months. The MP measurements showed a significantly greater loss of material than with Pro Seal. The surface was still sufficiently sealed after 6 months, which can be assigned to a mechanical load-bearing capacity (wear resistance) of Light Bond. The more than one-year durability of the sealant promised by the manufacturer is very likely. Light Bond showed good properties against mechanical stress in this study.

The technical literature contains numerous studies describing contrary results for ClinproXT Varnish. In 2015, Kumar Jena et al.^15^ investigated this sealant in patients undergoing early orthodontic treatment with fixed appliances regarding protection against the development of WSL. Compared to the untreated control group, significantly fewer WSL were observed in teeth sealed with ClinproXT Varnish. Mehta et al.^32^ examined 126 extracted premolars for WSL in their in vivo study from 2015. Except for three teeth, no demineralization lesions were detected on the sealed premolars. The sealant also achieved better results than in our study with regards to mechanical loading capacity. Deckers et al.^7^ described a material loss of 1.4% after a toothbrushing simulation of 6 weeks and a material loss of 1.9% after 6 months. This material loss is not comparable to the values in the current study. It is conceivable that the storage period between sealing and initial measurement was too long, which results in changes of the material properties. In this study, a higher material loss was observed for ClinproXT Varnish in comparison to Light Bond and Pro Seal. It was only possible to measure two of the eight samples, as the varnish layer was already very brittle during the initial measurement and began to detach from the HA surface. The other six samples could not be measured because an air gap was already initially present between the HA and the sealant. Due to the small number of samples, these values are not representative. The material loss was 36.8% (MP) and 51.1% (OP) after 6 weeks of simulated brushing time and 75.3% (MP) and 66.6% (OP) after 6 months of simulated brushing time. This result indicates that the material is susceptible to mechanical stress, even after a short period of time. According to the manufacturer, protection should be expected for 6 months. This was proven by the two measurable samples.

In 2015, Paschos et al.^4^ demonstrated a protective effect against demineralization in contrast to the untreated group, but not as effective as ProSeal. In their clinical study from 2013, Bechtold et al.^31^ found no difference in the protective abilities against demineralization between ProtectoCaF2 Nano and Light Bond. The results of Deckers et al.^7^ also demonstrate insufficient protection against mechanical stress. In their trials, material loss was 91.7% after 6 weeks and 93.9% after 6 months. In this study in the case of ProtectoCaF2 Nano and Fluor Protector, the entire coating layer was removed after a rendering period of only 6 weeks. The layer thickness was initially not measurable with the applied methods, as it was too low. Even under optimum in vitro conditions and after repeated application of the coating, no sufficient layer was formed to protect the surface adequately. It is conceivable that due to the low viscosity of these sealants, the material is penetrated in the pore-like structure of the HA discs. The results of ProtectoCaF2 Nano are also repeatedly confirmed in the literature.

Van der Linden and Dermaut^33^ were unable to demonstrate any significant protection against the development of WSL in combination with the glass ionomer cement in 1998. Bichu et al.^34^, on the other hand, showed the lowest average lesion depth in the group treated with Fluor Protector after exposure to the demineralization bath. The results of this study are in favor of demineralization protection by Fluor Protector. The in vivo study by Shafi^35^ also confirms that Fluor Protector reduces the risk of developing WSL during treatment with a fixed orthodontic appliance. Deckers et al.^7^ recorded a significant loss of material after mechanical loading in his study. This was 40.3% after 6 weeks and 64.5% after 6 months. Thus, the material loss was lower than with ProtectoCaF2 Nano. The mechanical load-bearing capacity of Fluor Protector was also insufficient in this study. This is partially confirmed in the literature.

The control group Tetric EvoFlow recorded a material loss of 12.8% (MP) and 15.1% (OP) after 6 weeks and 29.3% (MP) and 20.5% (OP) after 6 months. The material losses of the sealants can be summarized and ranked for abrasion resistance to mechanical loading: Pro Seal™ > Light Bond™ > Clinpro™ XT Varnish. The mechanical resistance of ProtectoCaF2 Nano and Fluor Protector cannot be conclusively assessed. The results of the study indicate that an effective pre-treatment, through acid etching of the surface, improve the mechanical stability of the sealants. Ideally, a sealant must be bond to a tooth surface without any moisture or contaminations; so that the sealant is able to penetrate the enamel–sealant interface.

## Conclusion

Only the Pro Seal and Light Bond sealants demonstrated sufficient resistance against the mechanical load and provided protection of the bracket environment for 6 months. From a methodological point of view, we can conclude that both methods (mechanical and optical profilometry) can be applied; the results are in good agreement.

## Data Availability

The data and materials used for analysis have been referenced in the text or tables of the paper.

## Abbreviations

HA: Hydroxyapatite
OP: Optical profilometry
MP: Mechanical profilometry
WSL: White spot lesions
